# Volatile Flavor Analysis in Yak Meat: Effects of Different Breeds, Feeding Methods, and Parts Using GC-IMS and Multivariate Analyses

**DOI:** 10.3390/foods13193130

**Published:** 2024-09-30

**Authors:** Hongqiang Li, Bin Xi, Shuqin Lin, Defu Tang, Yaqin Gao, Xiangmin Zhao, Jing Liang, Wanyun Yang, Jinlu Li

**Affiliations:** 1 College of Animal Science and Technology, Gansu Agricultural University, Lanzhou 730070, China; lhq136531597@163.com (H.L.); linsq@gsau.edu.cn (S.L.); tangdf@gsau.edu.cn (D.T.); 15393662656@163.com (X.Z.); 18298411081@163.com (J.L.); nbx1208@163.com (W.Y.); 2 Laboratory of Quality & Safety Risk Assessment for Livestock Products of Ministry of Agriculture, Lanzhou Institute of Husbandry and Pharmaceutical Sciences, Chinese Academy of Agricultural Sciences, Lanzhou 730050, China; xibin0707@163.com (B.X.); 13609312788@163.com (Y.G.)

**Keywords:** yak meat, GC-IMS, multivariate analysis, volatile flavor, VOCs

## Abstract

This study investigates the effects of breeds, feeding methods, and parts on the volatile flavor of yak meat. Gas chromatography–ion mobility spectrometry (GC-IMS) and multivariate analysis were used to analyze the volatile organic components (VOCs) in yak meat from various sources. A total of 71 volatile compounds were identified, 53 of which were annotated based on the GC-IMS database. These include 20 alcohols, 16 ketones, 10 aldehydes, four alkenes, one ester, one acid, and one furan. Using VOC fingerprinting and multivariate analysis, yak meats from different sources were distinctly categorized. Breed had the most significant impact on yak meat VOCs, followed by feeding method and then part. Six volatiles with a variable importance in projection value greater than one were identified as potential markers for distinguishing yak meat. This study offers insights into the flavor profile of yak meat from different sources and demonstrates the efficacy of GC-IMS and multivariate analysis in characterizing and discriminating meats.

## 1. Introduction

Yak (*Bos grunniens*) serves as an essential livestock in the Qinghai–Tibet Plateau and surrounding areas, providing meat, milk, and other products for survival. It also functions as the primary mode of transport for herdsmen, earning the nickname “boat of the plateau” [1,2]. Over 20 million yaks exist globally, with more than 95% residing in China [3,4]. Adapted to the harsh alpine conditions above 3000 m, yaks utilize the grassland resources of the plateau for animal production [5]. Their long-term adaptation to low oxygen and cold climates enhances the meat quality—high in protein, low in fat, and with a mellow flavor and rich nutrition [1,4]. Such qualities significantly strengthen disease resistance and improve cellular vitality and body function [1]. Yaks thrive in a clean, growth-promoting ecological environment minimally impacted by industrial activities, pesticides, or fertilizers [6]. The natural growth process and semi-wild grazing near water sources and plants yield meat with high nutritional value and superior taste, referred to as super beef [7]. Due to its excellence and rarity, yak meat is highly sought after in international markets but is often counterfeited for profit [1]. Consequently, developing techniques for traceability and authenticity verification of yak meat and milk is critical.

As a key quality index, flavor significantly influences consumer purchase intentions. The unique flavor of yak meat derives from volatile organic compounds (VOCs) such as aldehydes, alcohols, hydrocarbons, ketones, esters, acids, alkanes, aromatics, and others [8,9]. These components vary with the yak’s age. Calf yak meat typically exhibits a caramelized, sweet flavor, whereas adult meat features a robust, fatty taste [10]. Pi et al. [11] found that female Yushu yak meat has superior flavor quality. They identified five key volatile components differing by gender—cetyl aldehyde, nonyl aldehyde, 1-octene-3-ol, 4-phenyl-2-butanone, and 2,3-pentanedione. Additionally, the feeding method significantly affects flavor. Meat from grazing yaks, containing higher levels of aldehydes, ketones, alkanes, and heterocyclic compounds, offers a better taste than that from house-fed yaks [9]. Notably, only house-fed yak meat contains acids, which can impart an unpleasant sour taste and urinary odor, detracting from the flavor [12]. Other factors influencing flavor quality include the yak’s living environment and species [11,13].

Current methods for analyzing volatile components include gas chromatography–mass spectrometry (GC-MS), electronic nose (E-nose), and gas chromatography–olfactometry (GC-O), among other technologies [14,15]. GC-MS remains the standard in gas phase analysis, yet it is becoming increasingly cost- and resource-intensive due to high infrastructure demands and scarce resources like helium. The simpler and less demanding GC-IMS could replace several classical GC-MS applications [16]. Additionally, GC-IMS offers greater energy and resource savings, highlighting its potential as a green analytical technology (GAC) [17]. GC-IMS is advantageous for its rapid response, high sensitivity, simple operation, and low cost, allowing for the separation and detection of molecular ionization without sample pretreatment. It is widely used in drug detection, disease monitoring, and environmental protection and excels in food flavor analysis [18,19]. For instance, Liedtke et al. [20] first used laser desorption and GC-IMS to identify olive oil, proposing its application in quality control and adulteration analysis. Li et al. [21] used GC-IMS fingerprint identification with principal component analysis (PCA) to study volatile component changes in different types of milk during cold storage at 4 °C, aiding milk processing and storage. Rodríguez et al. [22] applied GC-IMS nondestructive testing combined with multivariate analysis to discriminate the geographical origin, curing plant, and commercial category of cured Iberian hams.

Studies on the characterization of VOCs in yak meat based on gender, feeding method, and processing show significant benefits [9,11,13,23]. They help consumers understand key flavor differences in yak meat from various sources, guiding deep processing for enterprises. However, the impact of various factors (such as breed, feeding method, part, etc.) on yak meat VOCs remains unclear. Which factors play a leading role in yak VOCs? What VOCs are potential markers used to distinguish yak meat? In addition, there are few reports about GC-IMS identification, fingerprint analysis, and flavor differentiation of yak meat VOCs.

In this study, GC-IMS was used to analyze VOCs from different yak meat breeds, feeding methods, and parts, exploring their effects on flavor characteristics and creating VOC fingerprints. Additionally, differential VOCs were identified using PCA and partial least squares discriminant analysis (PLS-DA). These findings support the sustainable use and quality processing of yak meat resources and offer a new strategy for flavor certification.

## 2. Materials and Methods

### 2.1. Ethics Statement

The animal study was approved by the Animal Care Committee at Gansu Agricultural University (approval number GAU-LC-2020-056). All animals in this study were approved by the Animal Ethics Committee of Gansu Agricultural University (approval number 2006-398, approval data 25 November 2020).

### 2.2. Sample Collection and Processing

**Figure 1:**
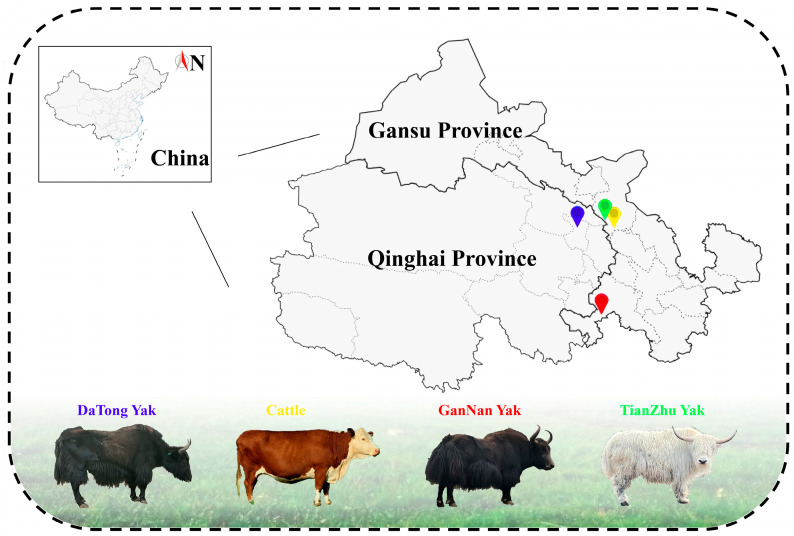
Distribution of sampling points in Gansu and Qinghai Province, China.

Samples were collected in September 2022. Information on the source and abbreviations of the meat samples is listed in Figure 1 and Table 1. Three adult male cattle, Tianzhu yak, Datong yak, and Gannan yak, similar in age (2 years), health status, and body shape, were selected. Group DTL-F was fed a total mixed ration (TMR) according to the Beef Cattle Feeding Standard (NY/T 815-2004), as shown in Table S1. Before the tests, all yaks were dewormed internally and externally. The DTL-F group received food twice daily and had continuous access to water. Other groups (CML, TZL-G, TZT-G, DTL-G, and GNL-G) grazed on natural pastures without human intervention.

Animals were slaughtered in local commercial abattoirs according to Chinese standard protocols (GB/T 19477-2004) and performed in accordance with animal welfare and ethics. After 24 h of cooling at 4 °C, samples of longissimus dorsi and triceps brachii were collected and cleansed of excess external fat and connective tissue. The muscle samples were vacuum-packed and transported to the laboratory under refrigerated conditions (4 °C), and stored at −80 °C for volatile compound identification.

### 2.3. Chemical and Reagents

For accurate retention index (RI) calculation of VOCs, C4–C9 methyl ketone was used as an internal standard, sourced from Sinopharm Chemical Reagent Beijing Co., Ltd. (China, Beijing). Ultrapure water was produced using a Jingqi ultrapure water system JQ-III-30. High-purity nitrogen (N_2_, 99.999%) was supplied by Air Liquide (Lanzhou, China).

### 2.4. GC-IMS System

The meat sample was homogenized (Mixer B-400, Buchi, Switzerland), and 3 g was placed in a 20 mL headspace vial for GC-IMS analysis (FlavourSpec^®^, G.A.S., Dortmund, Germany). The automatic injection settings were as follows: a headspace temperature of 60 °C for 15 min; a needle temperature of 85 °C; shaking at 500 rpm; a headspace injection volume of 500 μL; analysis with non-diversion mode. Chromatographic conditions were as follows: a column (FS-SE-54-CB-1, 15 m × 0.53 mm, 1.0 μm) at 60 °C and a total run time of 20 min. The flow rate started at 2 mL/min for 2 min, increased to 100 mL/min over 8 min, and was maintained for the remainder (10 min). Nitrogen, 99.999% pure, served as both carrier and drift gas; the IMS detector temperature was 45 °C, and the drift gas flow was set at 150 mL/min.

### 2.5. Data Processing and Statistical Analyses

VOCs from different meat sources were determined and identified using Laboratory Analytical Viewer (LAV) analysis software and Library Search qualitative software. Using n-ketones C4-C9 as an external standard reference, the RI of each compound was calculated [16]. Volatile compounds were identified by comparing the RI and drift time (Dt). The RI was searched using the National Institute of Standards and Technology (NIST) 17 library. Dt was searched using the GC-IMS library (G.A.S., Dortmund, Germany). The educed mobility *K*_*o*_ was also used for the compounds’ identification. *K*_*o*_ (cm^2^ V^−1^ s^−1^) is the normalized expression of ion mobility (*K*) in standard conditions of temperature and pressure (Equation (1)). *K* is a measure of friction linked to an observable, depends on the ion drift time taken to traverse the length of the mobility cell (Equation (2)) [24]. Based on peak intensity, volatile compounds were quantified by using GC-IMS LAV-Quantification software (version 2.2.1) [2]. Due to varying compound concentrations, some single compounds produced multiple signals or spots (dimers or trimers) [25]. The GC-IMS fingerprint was compared using the Gallery Plot plugin in LAV.

(1) $$K_{o}=K\times \frac{T_{o}}{T}\times \frac{p}{p_{o}}$$

(2) $$K=\frac{L}{E\times DT}$$

*T*, the temperature of the drift gas in *K*.

*T*_*o*_, the normal temperature: *T*_*o*_ = 273.16 *K*.

*P*, the pressure of the drift gas in kPa.

*p*_*o*_, the normal pressure: *p*_*o*_ = 101.3 kPa.

*L*, the length of the drift region in cm.

*E*, the electrical field strength in V/cm.

*DT*, the drift time in s.

Each sample was measured three times. The results were analyzed using SPSS version 22.0 (IBM, Armonk, NY, USA) with one-way analysis of variance (ANOVA). Differences were evaluated using Tukey’s test. Data with *p* < 0.05 were considered significant. PCA, PLS-DA, and cluster analysis were conducted using MetaboAnalyst software. Differential VOCs were identified based on a variable importance in projection (VIP) > 1.

## 3. Results and Discussion

### 3.1. Identification and Analysis of VOCs in Yak Meat Based on GC-IMS

**Figure 2:**
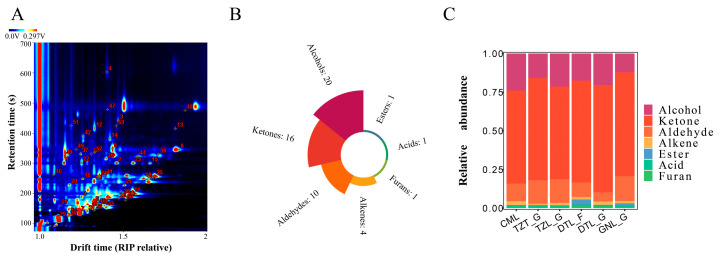
VOC profiles of yak meat from different sources. Number of VOCs (**A**), number of VOC categories (**B**), and percentages of VOC types (**C**).

To investigate the impact of different breeds, feeding methods, and parts on VOCs in yak meat, GC-MS was used to detect VOCs in all samples and analyze the differences. As shown in Figure 2A,B, and Table 2, a total of 53 VOCs were detected: 20 alcohols, 16 ketones, 10 aldehydes, four alkenes, one ester, one acid, and one furan. This count is notably higher than those identified using GC-MS and GC-IMS in previous studies [9,26]. This could be because the number of detected peaks in GC-IMS can vary widely depending on factors such as the type of sample, the chromatographic conditions, and the sensitivity of the instruments [16]. Additionally, it is important to note that cooked yak meat contains abundant VOCs, generated by the Maillard reaction between amino compounds and reducing sugars, lipid degradation, and lipid–Maillard interactions during heating [27,28].

To clearly illustrate differences in VOCs among samples, VOC peak volumes on the topographic representations of spectra were normalized (Figure 2A), and the relative content of volatile components in different samples was quantified. According to Figure 2C, aldehydes, alcohols, and ketones were the predominant VOCs in various yak meat samples. These compounds are also major flavor components in chicken meat [29]. Although alkanes are relatively abundant in yak meat [11], their contribution to meat flavor is minimal due to their high flavor threshold.

### 3.2. Effects of Different Feeding Methods on VOCs of Yak Meat

**Figure 3:**
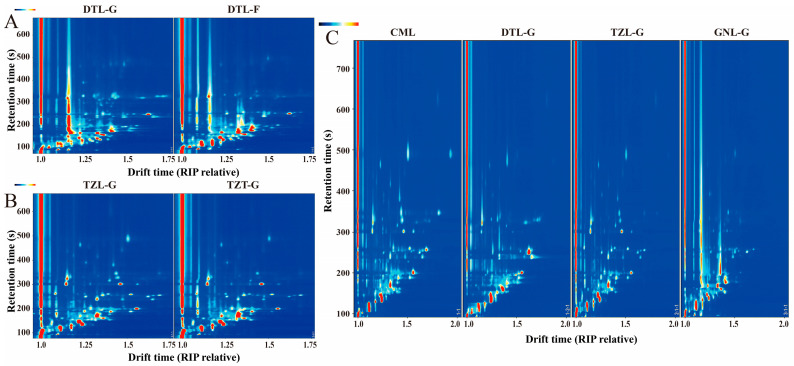
Comparison of VOCs in yak meat from different sources. Topographic representations of spectra for VOCs in yak meat from various feeding methods (**A**), parts (**B**), and breeds (**C**).

Topographic representations of VOC spectra in yak meat (DTL-G and DTL-F) under different feeding methods are depicted in Figure 3A. The vertical axis shows the gas phase retention time, and the horizontal axis shows the ion migration time. The background is blue, and the red vertical line on the left represents the reaction ion peak (RIP). Each point near the RIP indicates a volatile compound, with color variations representing concentration levels: white for lower, red for higher, and darker hues for the higher concentrations. As evident in Figure 3A, yak meats from traditional grazing (DTL-G) displayed more species and higher concentrations of VOCs compared to yard fattening (DTL-F), aligning with prior studies [9,12]. Grazing yaks benefit from extensive exercise and a diet rich in fatty acids, minerals, and antioxidants, resulting in a favorable fatty acid profile and enhanced flavor deposition [23,30].

To intuitively compare volatile compound differences in yak meat from various feeding methods, fingerprints of all VOCs from the spectra were generated automatically, as shown in Figure S1. Each row corresponds to yak meat samples from one feeding method, while each column shows the relative content of compounds in different samples. High-content VOCs in red areas, including seven ketones, five aldehydes, four alcohols, one acid, and one alkene, contribute to the robust flavor of naturally grazed yak meat. Among them, acids were detected only in the traditional grazing group (DTL-G), which is consistent with the results of a previous study [31], but this is controversial [12]. Ketones include 2,3-pentanedione, 2-nonanone M, 2-octanone, 2-hexanone, 2-nonanone D, 3-pentanone, and 3-octanone D, mostly with creamy or fruity aromas, which have a positive effect on meat flavor [32]. Aldehydes include 2-methyl propanal, nonanal, E 2-octenal M, E 2-heptenal, and E 2-octenal D. Most aldehydes come from oxidative hydrolysis of fats, and a very small portion of them come from the Meladic reaction of sugars [33]. Alcohols include 2-hexanol, 2-methyl butanol, 2-hexen-1-ol D, and 1-octanol, and they generally have a botanical aroma, rancid flavor, and chemicals. Limonene gives meat a lemon- and citrus-like flavor [34]. The content of ethyl acetate (aromatic, brandy) and 3-hydroxy-2-butanone (butter, creamy, and green pepper) in DTL-F was significantly higher than that in DTL-G. Moreover, VOC variation among individuals was more pronounced under natural grazing compared to in-house feeding. Based on the volatile compound data, similarity indices for the samples were calculated and are presented in Table S2, revealing that yak sample similarity (DTL-G and DTL-F) ranged from 48% to 80%. This indicates differences in VOCs among feeding methods, yet some similarities exist. In general, VOC composition was richer in yaks under grazing conditions but varied significantly among individuals.

After analyzing the flavor compounds, the current study found some noteworthy results that merit discussion. Although the flavor profile of grazing yaks was superior, the meat quality from fattening yaks excelled (shear force and pH) [35,36]. However, due to differences in rumen microbial diversity, the muscle fatty acid composition in fattening yaks was inferior compared to grazing yaks [12]. Wang et al. [35] discovered that in the longissimus dorsi muscle of Ashdan yaks, specific genes (*ACTA1* and *FBXO32*) and metabolites (L-carnitine and myostatin) are involved in meat quality regulation. Furthermore, grazing yak meat quality varies with grass phenology. Grass in different phenology periods (regreen and hay periods) was associated with modified amino acids and fatty acids composition of yak meat as well as altered regulation of biological pathways, which was correlated with changes in rumen bacterial communities [37].

### 3.3. Effects of Different Parts on VOCs of Yak Meat

Figure 3B displays the spectral topography for VOCs in different parts of yak meat (TZL-G and TZT-G). Although the VOC compositions of TZL-G and TZT-G are similar, the TZL-G samples have higher concentrations of certain volatiles. From the fingerprint spectrum (Figure S2), VOCs in the red region, such as 1-octen-3-ol, 5-methyl-2-furanmethanol, E 2-heptenal, E 2-octenal, nonanal, 3-methylbutanal, heptanal, and phenylacetaldehyde, are abundant in TZL-G. The similarity of VOC compositions between the different yak meat parts was calculated, and the results are presented in Table S3. It was found that the similarity of different parts of yak meat (TZL-G and TZT-G) is high, indicating small differences in VOC composition.

Muscles in different parts of the cattle carcass exhibit distinct characteristics due to variations in muscle fiber and connective tissue. Leg muscles typically have high daily activity, strong toughness, and lower meat quality. In contrast, meat from areas away from the legs is tenderer and of higher quality [38,39]. Research has shown that the histidine metabolism pathway affects VOC formation in various yak muscle tissues [26]. Further, analysis of flavor components in different parts of Yushu yak meat revealed similar flavors in the psoas major, transverse chest, intercostal, and arm muscles, while they differed in semimembranosus muscle [32].

### 3.4. Effects of Different Breeds on VOCs of Yak Meat

Topographic representations of spectra for VOCs in yak meat from different breeds are depicted in Figure 3C. The results show that the VOC composition and content vary among longissimus dorsi muscle samples from different breeds, presenting distinct flavor characteristics. The fingerprint spectrum shows that the VOC composition of CML and TZL-G is similar (Figure S3). However, CML contains higher levels of certain VOCs, such as E-2-octenal, E-2-heptenal, nonanal, 2-hexen-1-ol, 2-octanol, 2-ethyl-1-hexanol, 5-methyl-2-Furanmethanol, and octanoic acid. Similarities in VOC composition were also noted between the GNL-G and DTL-G samples. Using the VOC data, similarity indices for the samples were calculated and are listed in Table S4. It was found that the similarity between CML and the three yak samples (TZL-G, GNL-G, and DTL-G) was low, but high among the three yak samples themselves.

Previous studies have indicated that VOCs in meat depend on breed [29,40]. Our study confirms that yak meat VOCs are also breed-dependent. Yak meat, rich in nutrients and essential amino acids, offers a unique flavor. However, due to the special environmental conditions and predominant grazing lifestyle of yaks, yak meat tends to be less tender than meat from other cattle breeds [35]. While cattle, Tianzhu yak, Datong yak, and Gannan yak all belong to the genus Bovine, they exhibit distinct flavor profiles, possibly due to environmental and genetic differences [41,42], leading to intra-genus VOC variability. A recent study identified phenotypic variations in domestic yaks due to extensive genetic introgression, explaining the origin of the Tianzhu yak as a new mutation from cattle introgression [5]. This genetic background contributes to the flavor similarity between CML and TZL-G.

### 3.5. Multivariate Analysis of VOCs

**Figure 4:**
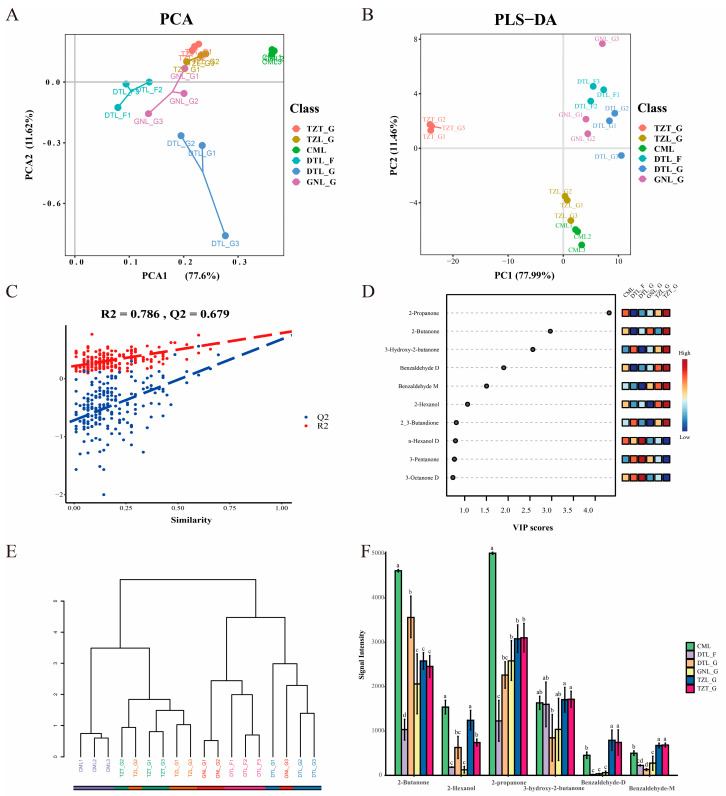
Multivariate analysis of VOCs in yak meat from different sources. Principal component analysis (PCA) (**A**), partial least squares discriminant analysis (PLS-DA) (**B**) score plots based on flavoromics data, corresponding PLS-DA validation plots (**C**), differential VOCs between yak meats from different sources (VIP > 1) (**D**), and cluster analysis (**E**) and signal intensity (**F**) of differential VOCs. Statistical analysis was performed using one-way ANOVA. Different letters indicate significant differences (*p* < 0.05).

GC-IMS combined with multivariate analysis can be a convenient and powerful method for characterizing different meats [22,29,43]. PCA, a data dimensionality reduction technique, can effectively reveal relationships between samples while retaining the most impactful features. In this study, signal intensity data from 53 flavor components were subjected to PCA. Principal component scores were ranked by contribution rate, and the first two were analyzed visually. As depicted in Figure 4A, PC1 contributes 77.6%, PC2 adds 11.62%, and the cumulative contribution rate post-reduction is 89.22%, effectively representing the original data characteristics. In the PC1 direction, CML was distinct from other samples, confirming the ability of VOCs to differentiate cattle from yak meat. In the PC2 direction, TZT-G and TZL-G were grouped, as were GNL-G, DTL-F, and DTL-G. Surprisingly, we found that DTL-F and GNL-G exhibited similar volatile flavors. The results showed that the composition of VOCs in yak meat could be used to distinguish the breed, feeding method, and part of yak, and the impact of the three factors on yak meat VOCs was as follows: breed > feeding method > part.

PLS-DA, a supervised analysis method based on partial least squares regression, aids in visualizing, discriminating, and predicting complex data. To identify VOCs for discriminating yak meat from different sources, we conducted PLS-DA on the dataset. Different meat sources were distinctly separated (Figure 4B). To verify the reliability of the model, cross-validation and permutation tests were applied to the R2 and Q2 values. The cross-validation of the study showed R2 at 0.786 and Q2 at 0.679, confirming the validity of the model for category differentiation since Q2 > 0.5 (Figure 4C). No overfitting was evident in 200 permutation tests, proving the predictive and explanatory strength of the model.

Contributions of variables to classification were quantified by the VIP values in the PLS-DA model, and VOCs with a VIP value > 1 were identified as potential characteristic volatile flavor components [44]. According to Figure 4D, six VOCs had a VIP > 1: 2-propanone, 2-butanone, 3-hydroxy-2-butanone, benzaldehyde-D, benzaldehyde-M, and 2-hexanol. Using these six components, hierarchical clustering analysis (Figure 4E) grouped CML, TZT-G, and TZL-G into one category, while DTL-F, DTL-G, and GNL-F were classified together, aligning with the PCA results. These outcomes suggest that multivariate analysis can be applied to distinguish different samples and identify biomarkers in GC-IMS data.

Among the six VOCs, the top three by VIP value were ketones, which, except for 3-hydroxy-2-butanone, were more abundant across samples, particularly in CML (Figure 4F). Ketones form in meat products through chemical (autooxidation) or enzymatic (β-oxidation) processes from fatty acids [45]. Reports on the specific flavor profile of 2-propanone in meat are limited; it has a pungent, slightly sweet odor. As a characteristic flavor compound in fermented meat products, 2-butanone exhibits a fragrant, fruity, pleasant odor [46,47]. It is also found in vegetables and dairy products [21,48]. 3-hydroxy-2-butanone, also known as acetoin, has a pleasant milky flavor [49] and results from the Maillard reaction, possibly from oleic acid oxidation or glucose decomposition [50]. Due to varying concentrations, some compounds might appear as multiple signals or spots in GC-IMS (dimers or trimers) [25]. Benzaldehyde, known for its almond flavor, enhances the aroma of meat products. Its production may relate to fat accumulation and unsaturated fatty acid deposition in yak meat [51]. Research indicates that compounds like phenylpyruvic acid, phenylacetaldehyde, and β-phenylethylamine formed by the carbonylamine reaction are degraded into phenylacetic acid and benzaldehyde under free radicals from lipid peroxidation of 13-hydroperoxide and 4-oxononenal of linoleic acid [52]. The concentration of 2-hexanol was lowest in DTL-F and GNL-G; it forms through polyunsaturated fatty acid oxidation [53] and has a chemical, winey odor.

## 4. Conclusions

In this study, a comprehensive and comparative volatile analysis was performed to characterize and distinguish yak meat from different breeds, feeding methods, and parts. Overall, 53 VOCs across seven compound categories were identified. The primary volatile components in yak meat were ketones, alcohols, and aldehydes. Using VOC fingerprints and multivariate analysis, it was found that grazing yaks had a richer but more variable VOC composition than fattening yaks. Longissimus dorsi contained more VOCs than triceps brachii. The VOC composition was similar between the GNL-G and DGL-F samples. Overall, breed had the greatest influence on the volatile flavor of yak meat, followed by feeding method and then part. Additionally, six VOCs with a VIP > 1 were identified as potential markers for distinguishing yak meat.

Despite these findings, we are aware that our research has some limitations. It was somewhat biased because we limited our subjects of different feeding methods to Datong yak and only characterized VOCs in the longissimus dorsi and triceps brachii of Tianzhu yak. Therefore, our future research will incorporate a broader range of yak breeds, ages, feeding patterns, and types of tissues. We aim to establish a comprehensive VOC database for diverse yak breeds based on GC-MS, GC-IMS, GC-O-MS, and other multi-group learning segments. This will provide the theoretical foundation and technical support essential for enhancing yak meat quality and flavor.

## Figures and Tables

**Table 1:** Sample information.

Samples			Sample Collection Site	Sample Abbreviation
Breed	Feeding Mode	Part		
Cattle	Traditional grazing	Longissimus dorsi	Tianzhu Tibetan Autonomous County, Gansu Province, China	CML
Tianzhu yak	Traditional grazing	Longissimus dorsi	Tianzhu Tibetan Autonomous County, Gansu Province, China	TZL-G
Tianzhu yak	Traditional grazing	Triceps brachii	Tianzhu Tibetan Autonomous County, Gansu Province, China	TZT-G
Datong yak	Traditional grazing	Longissimus dorsi	Datong Hui Tu Autonomous County, Qinghai Province, China	DTL-G
Datong yak	Yard fattening	Longissimus dorsi	Datong Hui Tu Autonomous County, Qinghai Province, China	DTL-F
Gannan yak	Traditional grazing	Longissimus dorsi	Luqu County, Gansu Province, China	GNL-G

**Table 2:** Information on VOCs in yak meat.

Count	Compound	CAS#	Formula	MW	RI ^a^	Rt ^b^ [sec]	Dt ^c^
1	Benzaldehyde M	C100527	C_7_H_6_O	106.1	950.3	301.553	1.1466
2	Benzaldehyde D	C100527	C_7_H_6_O	106.1	950.3	301.553	1.4605
3	Nonanal	C124196	C_9_H_18_O	142.2	1073.8	442.575	1.472
4	Octanoic acid	C124072	C_8_H_16_O_2_	144.2	1166.1	603.03	1.4047
5	2-Octanol M	C123966	C_8_H_18_O	130.2	999.2	348.832	1.4364
6	2-Octanol D	C123966	C_8_H_18_O	130.2	996.6	346.061	1.8356
7	2-Butanone	C78933	C_4_H_8_O	72.1	582.7	137.805	1.2399
8	3-Hydroxy-2-butanone	C513860	C_4_H_8_O_2_	88.1	712.4	170.092	1.3268
9	1-Pentanol M	C71410	C_5_H_12_O	88.1	763	188.268	1.2511
10	1-Pentanol D	C71410	C_5_H_12_O	88.1	763	188.268	1.5226
11	2-Hexanol	C626937	C_6_H_14_O	102.2	793.2	201.097	1.5571
12	E 2-octenal M	C2548870	C_8_H_14_O	126.2	1055.4	416.605	1.3286
13	E 2-octenal D	C2548870	C_8_H_14_O	126.2	1054.3	415.083	1.8143
14	2-Ethyl-1-hexanol	C104767	C_8_H_18_O	130.2	1028	381.602	1.4216
15	E 2-heptenal	C18829555	C_7_H_12_O	112.2	949.6	300.943	1.6625
16	n-Hexanol M	C111273	C_6_H_14_O	102.2	867.5	240.478	1.3265
17	n-Hexanol D	C111273	C_6_H_14_O	102.2	866.5	239.904	1.6482
18	2-Heptanone M	C110430	C_7_H_14_O	114.2	881.5	249.408	1.2594
19	2-Heptanone D	C110430	C_7_H_14_O	114.2	882.8	250.234	1.6241
20	2-Hexen-1-ol M	C2305217	C_6_H_12_O	100.2	847.5	228.688	1.1788
21	2-Hexen-1-ol D	C2305217	C_6_H_12_O	100.2	845.8	227.725	1.5074
22	2-Furanmethanol, 5-methyl- M	C3857258	C_6_H_8_O_2_	112.1	951.9	302.963	1.252
23	2-Furanmethanol, 5-methyl- D	C3857258	C_6_H_8_O_2_	112.1	950.3	301.54	1.5611
24	Heptanal M	C111717	C_7_H_14_O	114.2	892.6	256.838	1.3582
25	Heptanal D	C111717	C_7_H_14_O	114.2	893.2	257.253	1.6918
26	Heptanol	C53535334	C_7_H_16_O	116.2	965.7	315.413	1.3998
27	2-Pentanone M	C107879	C_5_H_10_O	86.1	679.9	160.307	1.1229
28	2-Pentanone D	C107879	C_5_H_10_O	86.1	679.9	160.307	1.3615
29	2-Propanone	C67641	C_3_H_6_O	58.1	467.2	120.6	1.1122
30	2-Methyl butanol	C137326	C_5_H_12_O	88.1	747.8	182.416	1.4714
31	2-Methylpropanal	C78842	C_4_H_8_O	72.1	599.7	141.099	1.2841
32	Ethyl acetate	C141786	C_4_H_8_O_2_	88.1	617.3	144.785	1.327
33	3-Methylbutanal	C590863	C_5_H_10_O	86.1	658.7	154.593	1.3995
34	2,3-Butandione	C431038	C_4_H_6_O_2_	86.1	559.2	133.611	1.1757
35	2-Propanol	C67630	C_3_H_8_O	60.1	492.2	123.659	1.1756
36	2-Hexanone	C591786	C_6_H_12_O	100.2	779.7	195.191	1.4952
37	2-Pentylfuran	C3777693	C_9_H_14_O	138.2	982.8	331.937	1.2456
38	3-Octanone M	C106683	C_8_H_16_O	128.2	981.9	330.974	1.3094
39	3-Octanone D	C106683	C_8_H_16_O	128.2	981.2	330.325	1.7121
40	Methional M	C3268493	C_4_H_8_OS	104.2	899.9	261.921	1.0858
41	Methional D	C3268493	C_4_H_8_OS	104.2	898.3	260.793	1.393
42	Phenylacetaldehyde	C122781	C_8_H_8_O	120.2	1035.1	390.305	1.2617
43	1-Octen-3-ol M	C3391864	C_8_H_16_O	128.2	974.8	324.035	1.1514
44	1-Octen-3-ol D	C3391864	C_8_H_16_O	128.2	974.4	323.677	1.5896
45	2,3-Pentanedione	C600146	C_5_H_8_O_2_	100.1	651.5	152.753	1.3066
46	3-Pentanone	C96220	C_5_H_10_O	86.1	693.7	164.311	1.3493
47	2-Nonanone M	C821556	C_9_H_18_O	142.2	1097.4	478.677	1.408
48	2-Nonanone D	C821556	C_9_H_18_O	142.2	1095.5	475.696	1.8718
49	Limonene	C138863	C_10_H_16_	136.2	997	346.505	1.2193
50	3-Methylbutan-1-ol	C123513	C_5_H_12_O	88.1	736.3	178.199	1.5133
51	Alpha-Methylbenzenemethanol	C98851	C_8_H_10_O	122.2	1061.4	424.826	1.189
52	2-Octanone	C111137	C_8_H_16_O	128.2	987.3	336.387	1.3295
53	1-Octanol	C111875	C_8_H_18_O	130.2	1060.8	423.97	1.4614

Note: a—the retention index calculated using n-ketones C4–C9 as the external standard on an FS-SE-54-CB column; b—the retention time in the capillary GC column; c—the drift time in the drift tube; M represents “Monomer”, and D represents “Dimer”.

## Data Availability

The original contributions presented in this study are included in the article/Supplementary Material; further inquiries can be directed to the corresponding author.

## Supplementary Materials

The following supporting information can be downloaded at: https://www.mdpi.com/article/10.3390/foods13193130/s1, Figure S1: Fingerprints of gallery plots for VOCs in yak meat from different feeding methods. Note: the numbers represent compounds that have not been identified. Figure S2. Fingerprints of gallery plots for VOCs in yak meat from different parts. Note: the numbers represent compounds that have not been identified. Figure S3. Fingerprints of gallery plots for VOCs in yak meat from different breeds. Note: the numbers represent compounds that have not been identified. Table S1: Composition and nutritional level of the TMR (air-dry basis)%. Table S2: Similarity between the different feeding methods of yak meat samples (%). Table S3: Similarity between the different parts of yak meat samples (%). Table S4: Similarity between the different breeds of yak meat samples (%).
