# Supplementary material for: Volatile Flavor Analysis in Yak Meat: Effects of Different Breeds, Feeding Methods, and Parts Using GC-IMS and Multivariate Analyses
Source: Foods. 2024 Sep 30;13(19):3130. doi: 10.3390/foods13193130 (PMC11476270; doi:10.3390/foods13193130)
Supplement: Supplementary file 1 [file foods-13-03130-s001.zip › Supplementary Figures.pdf]

Supplementary Material

Supplementary Figures

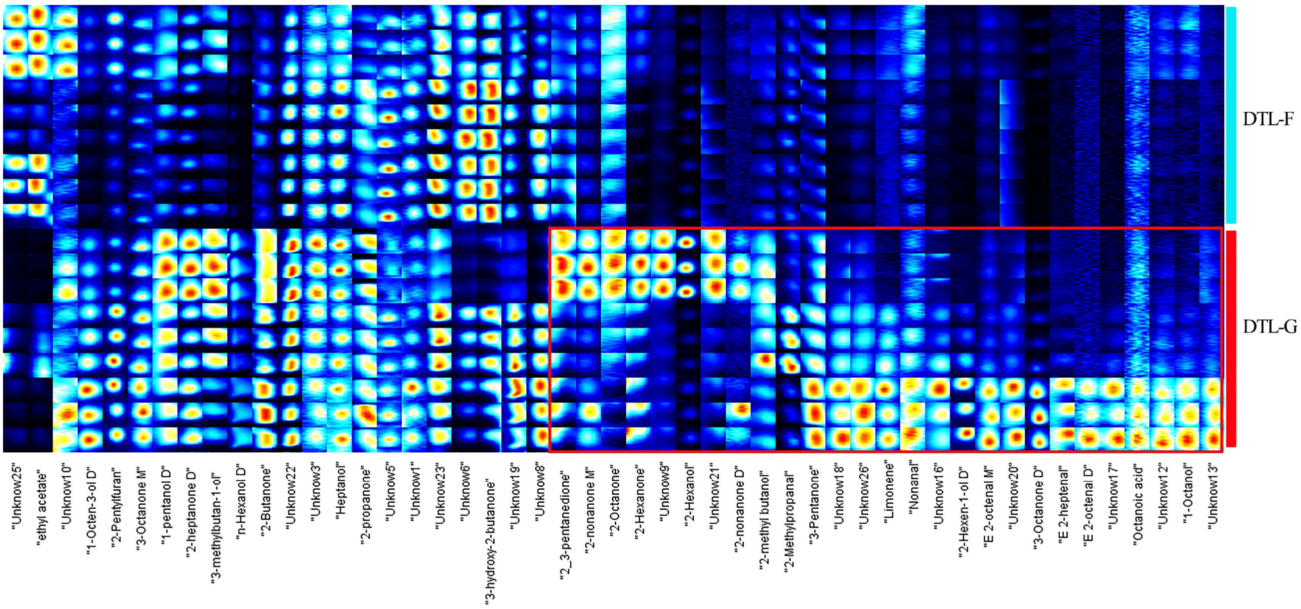

Figure S1. Fingerprints of gallery plots for VOCs in yak meat from different feeding methods. Note: the numbers represent compounds that have not been identified.

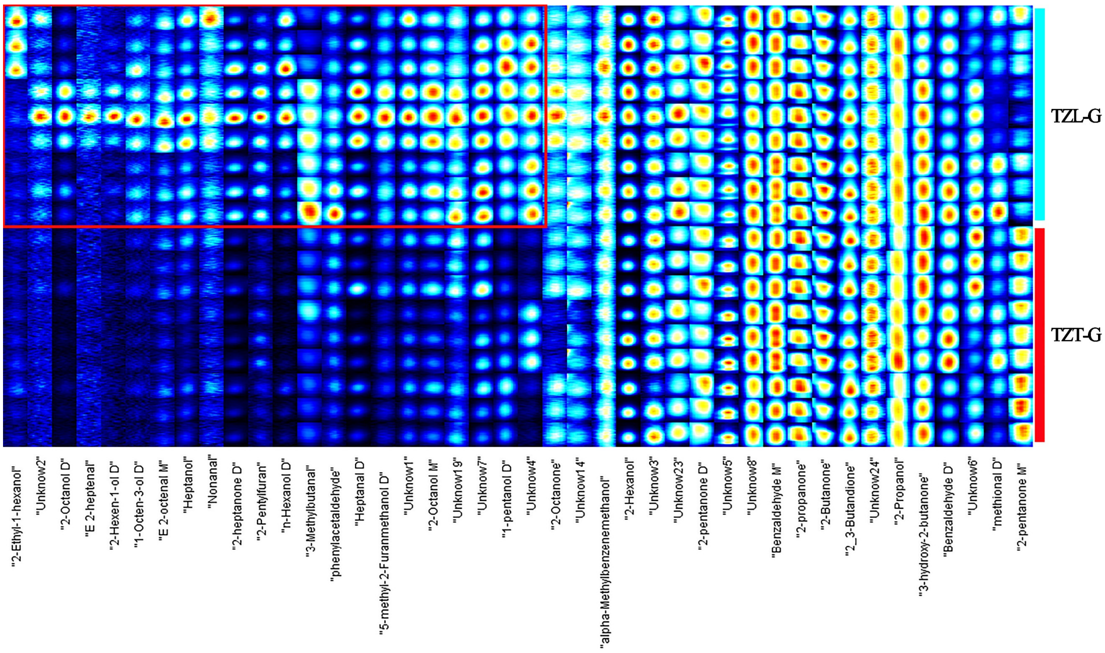

Figure S2. Fingerprints of gallery plots for VOCs in yak meat from different parts. Note: the numbers represent compounds that have not been identified.

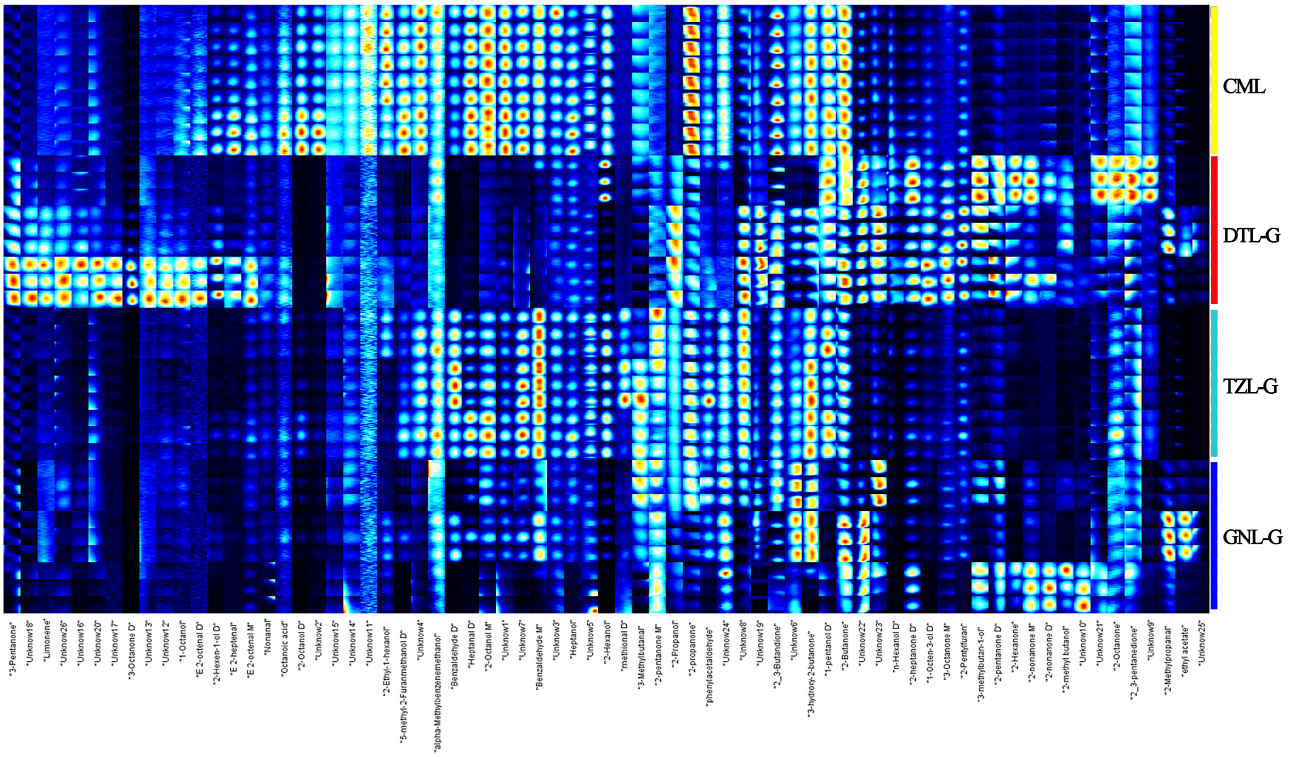

Figure S3. Fingerprints of gallery plots for VOCs in yak meat from different breeds. Note: the numbers represent compounds that have not been identified.
